# Supplementary material for: Dissecting Molecular Heterogeneity of Circulating Tumor Cells (CTCs) from Metastatic Breast Cancer Patients through Copy Number Aberration (CNA) and Single Nucleotide Variant (SNV) Single Cell Analysis
Source: Cancers (Basel). 2022 Aug 14;14(16):3925. doi: 10.3390/cancers14163925 (PMC9405921; doi:10.3390/cancers14163925)

Figure S4. Copy Number aberration (CNA) plots of the most representative circulating tumor cells (CTCs) for each patient (except for patient CH28). In the x-axis the chromosome number is reported, while the number of copies is reported in the y-axis. Black dots: normal copy number; blue dots: loss; red dots; gain.

Patient CH29

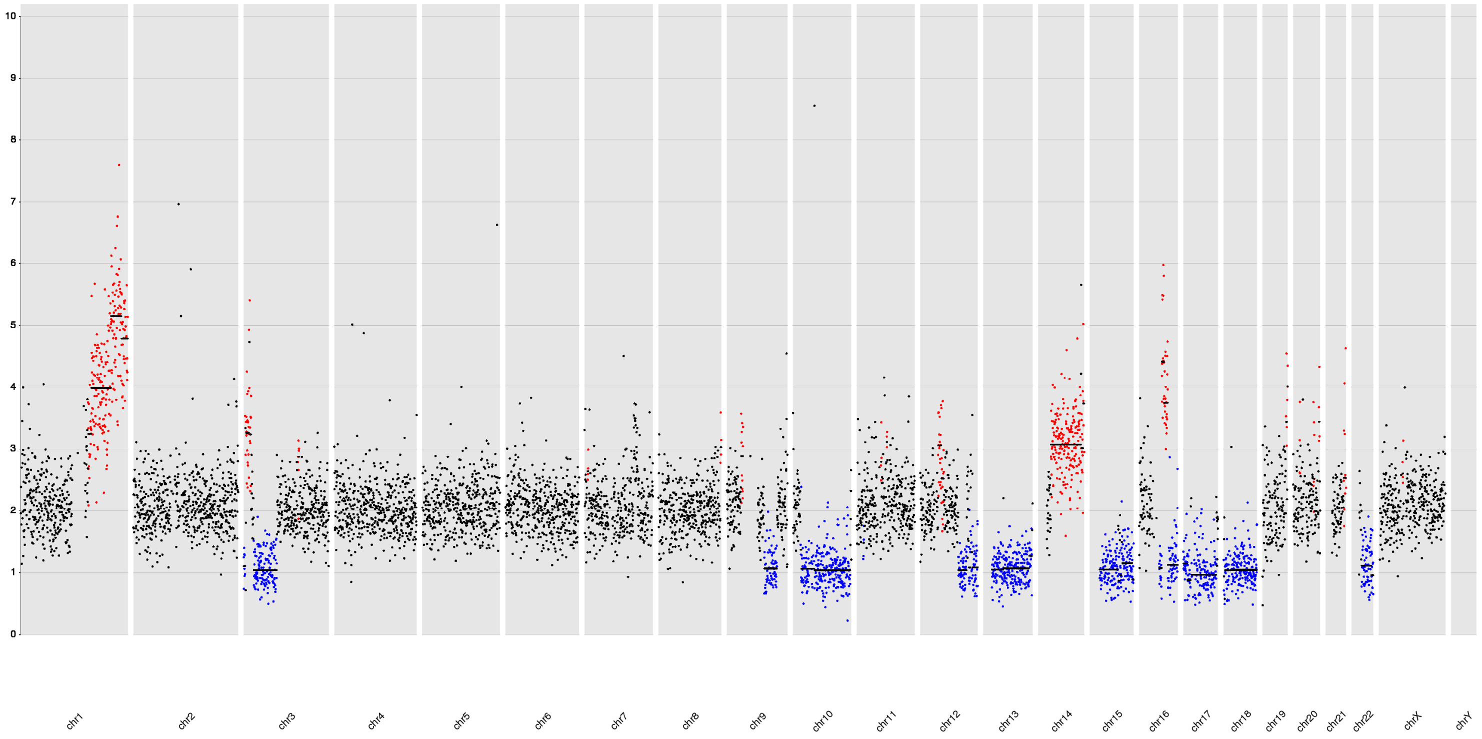

Patient CH30

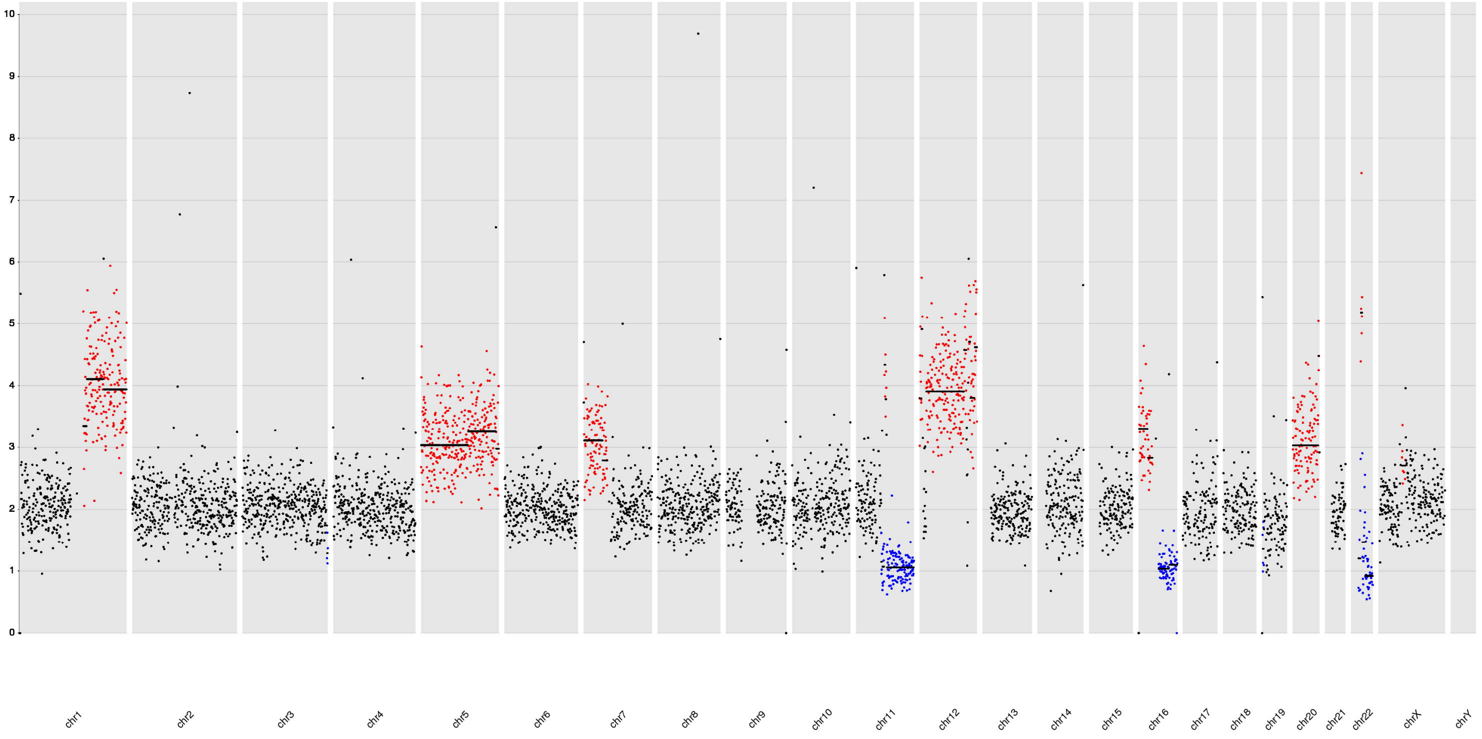

Patient CH32

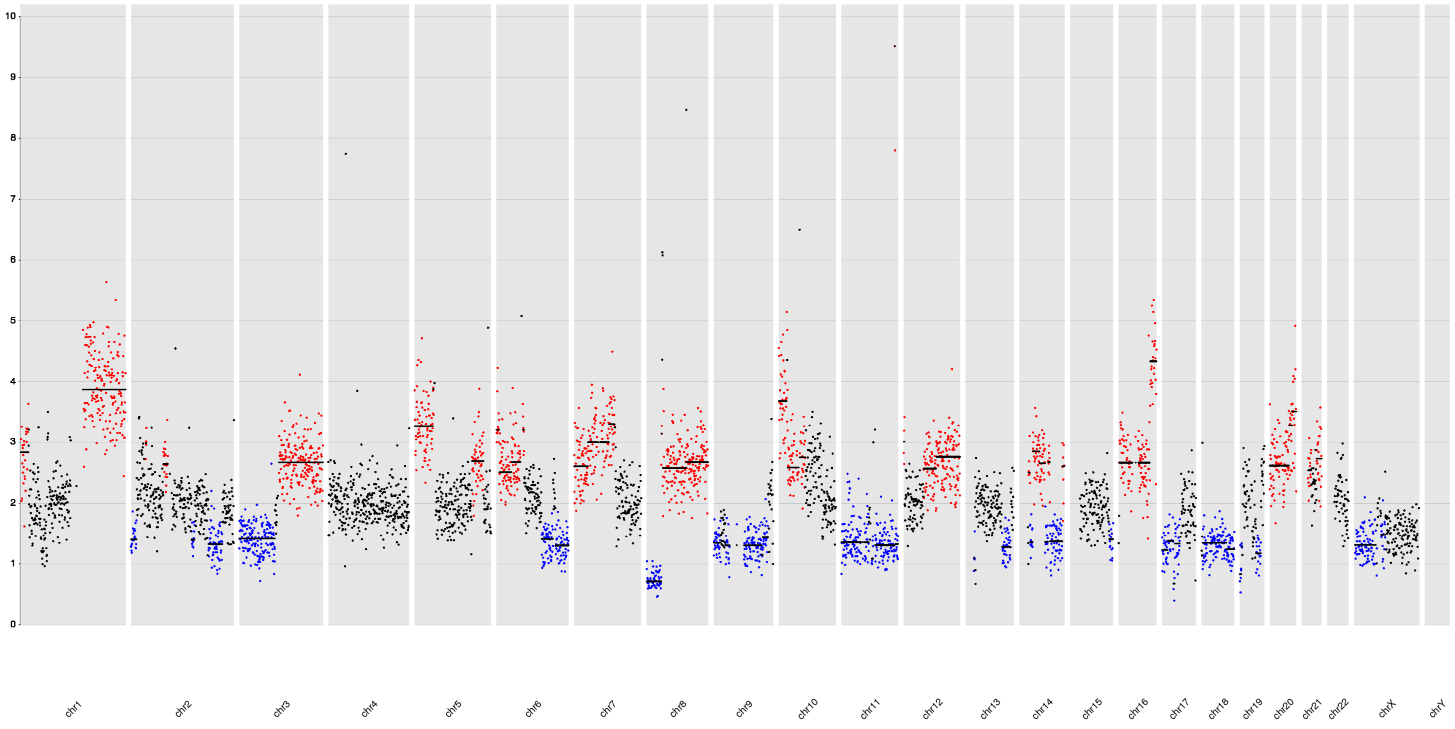

Patient CH46

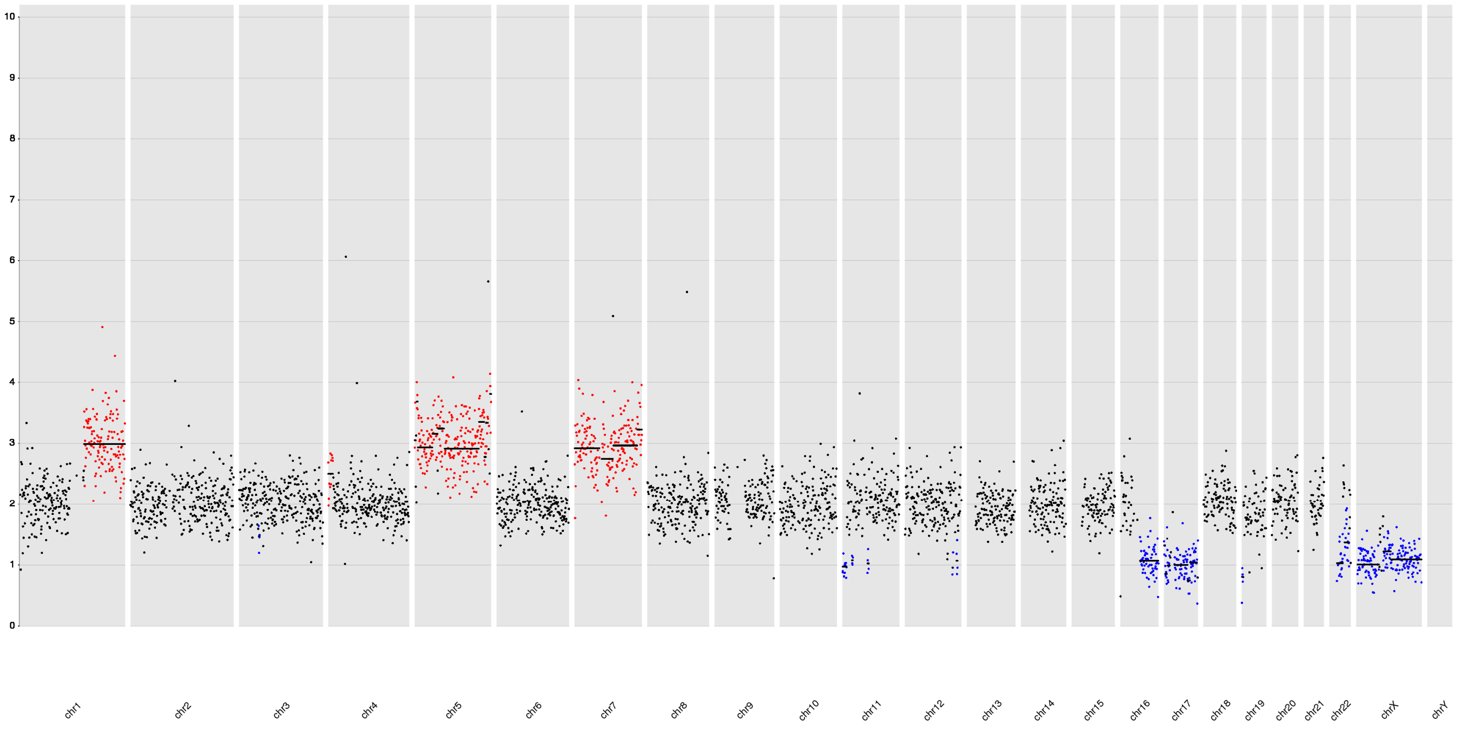

Patient CH47

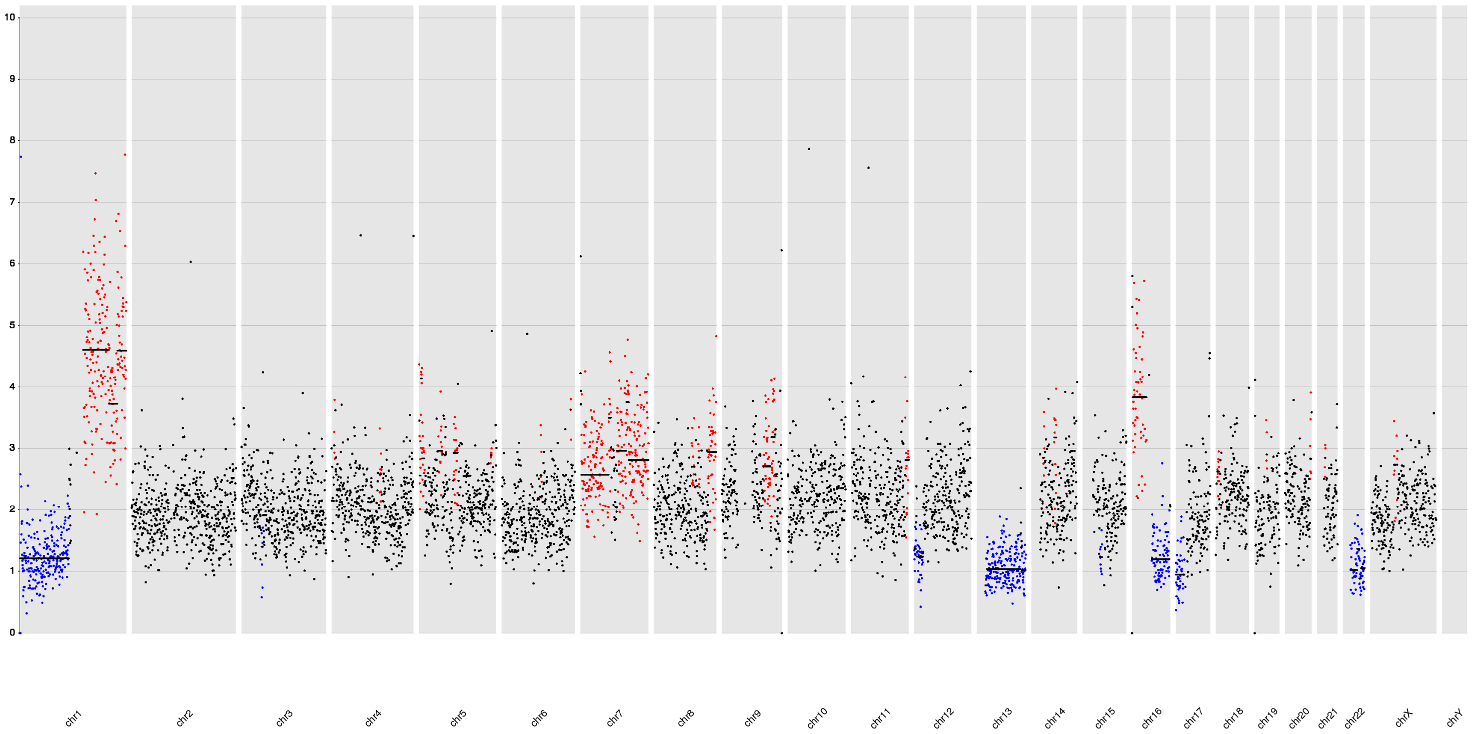

Supplement: Supplementary file 1 [file cancers-14-03925-s001.zip › Supplementary Material Figure S4.pdf]
