# Supplementary material for: Dissecting Molecular Heterogeneity of Circulating Tumor Cells (CTCs) from Metastatic Breast Cancer Patients through Copy Number Aberration (CNA) and Single Nucleotide Variant (SNV) Single Cell Analysis
Source: Cancers (Basel). 2022 Aug 14;14(16):3925. doi: 10.3390/cancers14163925 (PMC9405921; doi:10.3390/cancers14163925)
Supplement: Supplementary file 1 [file cancers-14-03925-s001.zip › Supplementary Material File S1.pdf]

Supplementary material File S1.

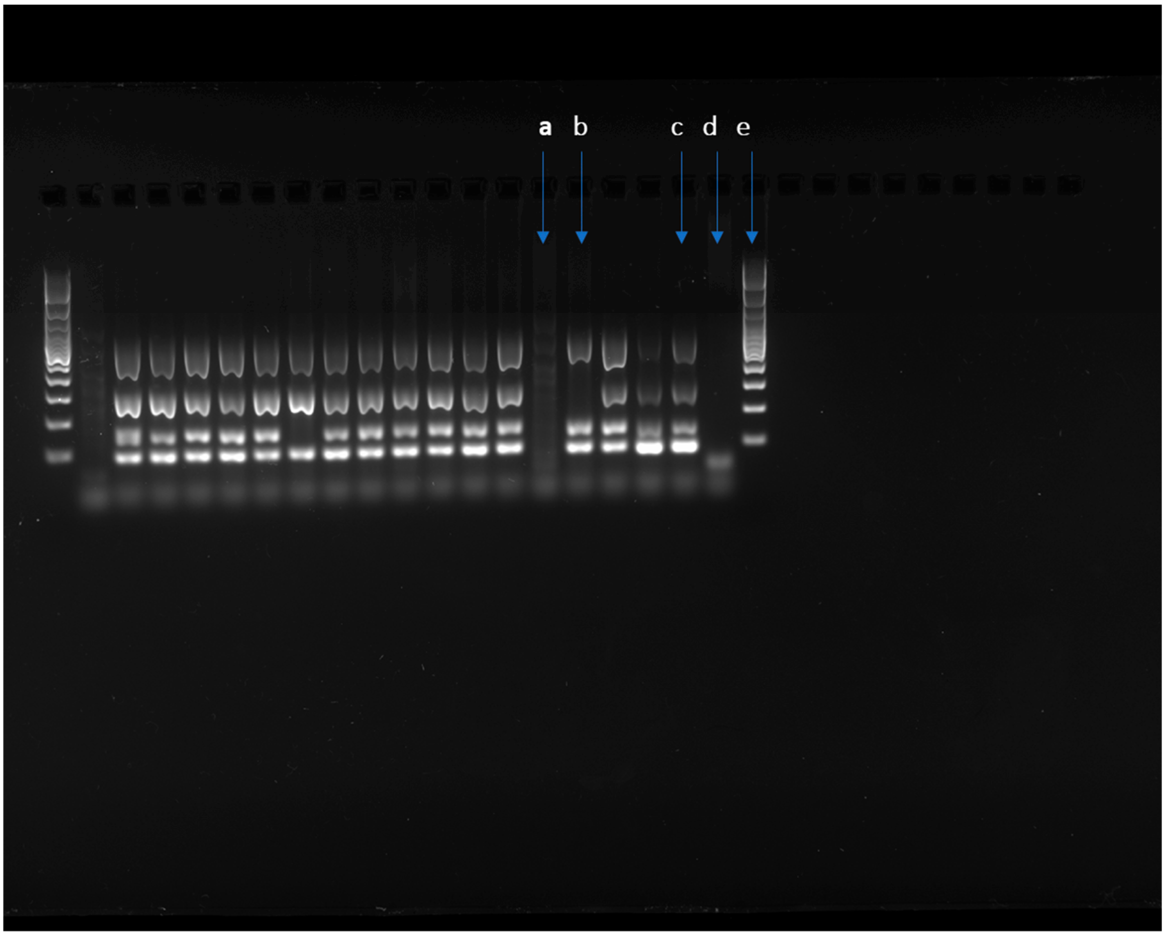

Figure S1. DNA integrity testing in sorted cells. Multiplex PCR was performed and four PCR products of different lengths from four chromosomal regions were amplified. The number of bands obtained by each PCR product was visualized on a 2% agarose gel through ultraviolet light. Each lane represents a single cell/pool. a: sample with no amplification (0 bands); b: sample with amplification of 3 targets (3 bands); c: positive control with amplification of 4 targets (4 bands); d: negative control; e: 100 bp marker.

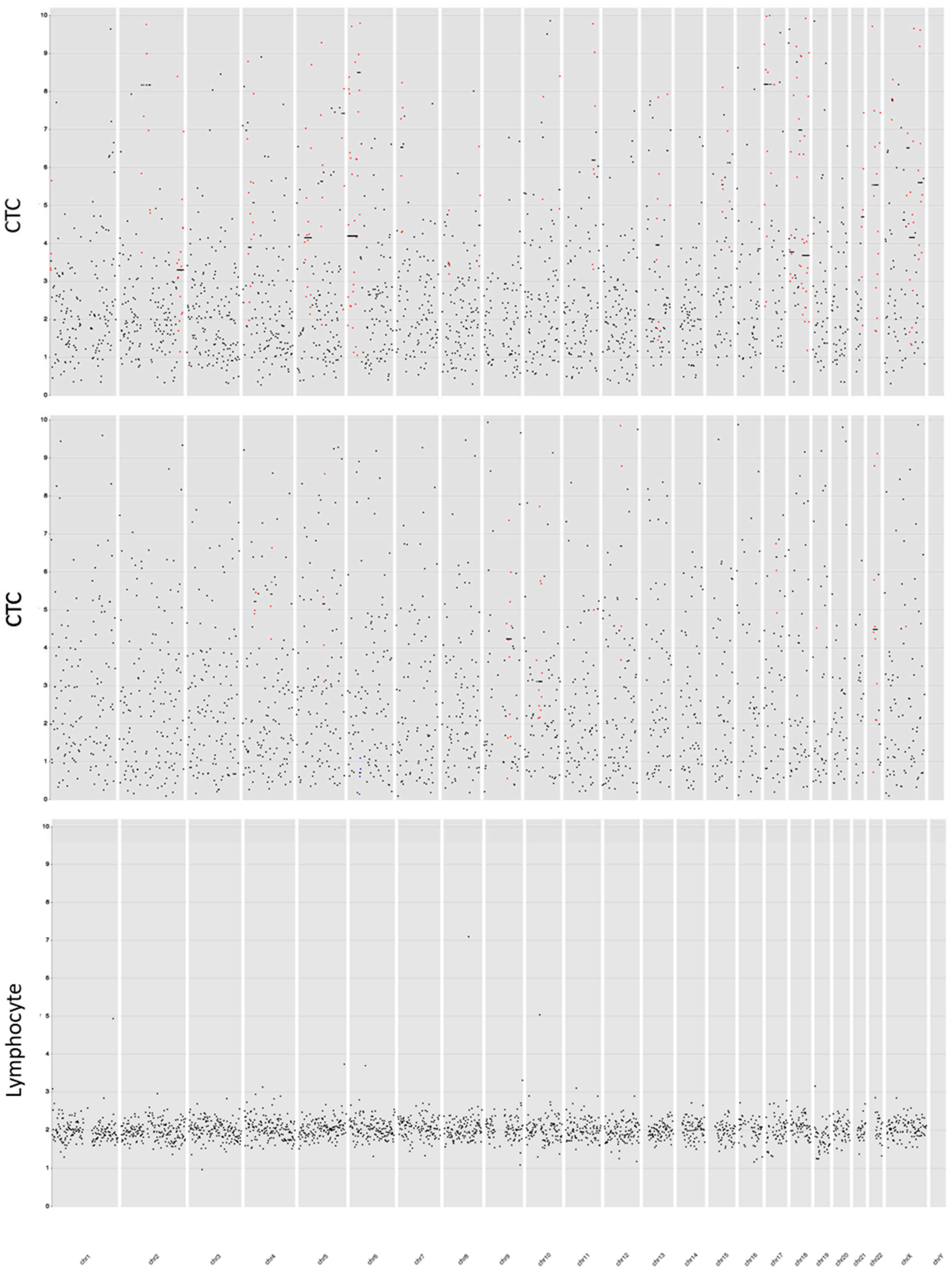

Figure S2. Genomic copy number aberration profiles of two representative single circulating tumor cells (CTCs) and one lymphocyte pool from patient CH28. While the lymphocyte sample displays an adequate profile, the CTC profiles are mainly characterized by background suggesting an apoptotic status.

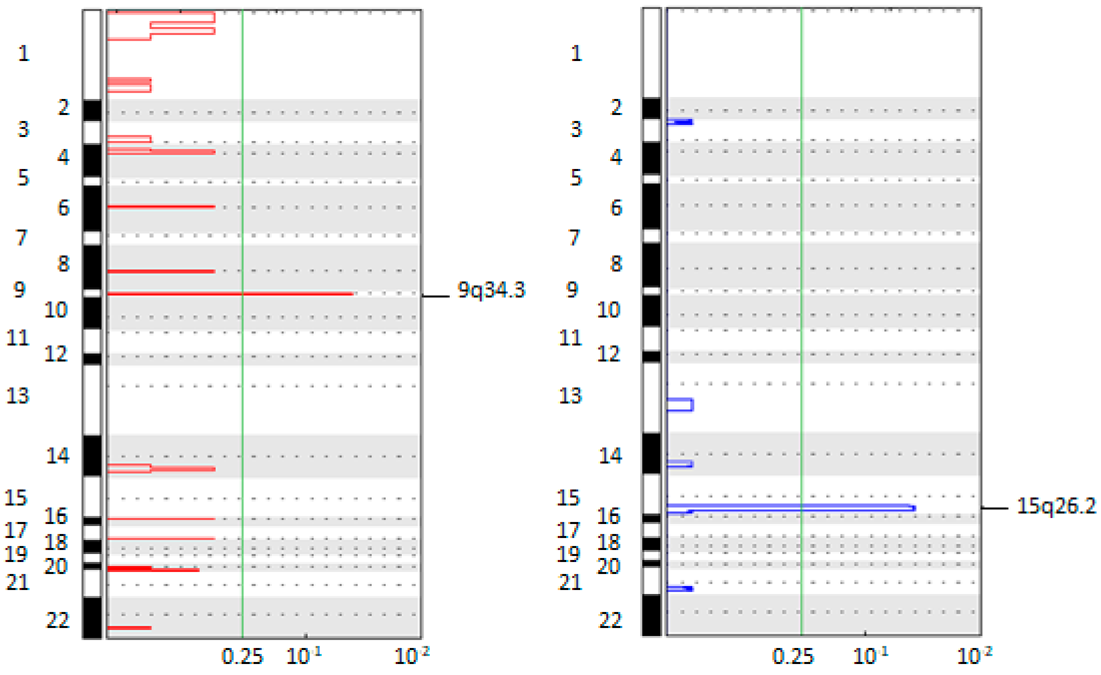

Figure S3. Genomic Identification of Significant Targets in Cancer (GISTIC) amplification and deletion plots on single circulating tumor cells (CTCs) from the crossed-patients CTC set. The genome is oriented vertically from top to bottom, and the GISTIC *q*-values at each locus are plotted from left to right on a log scale. The green line represents the significance threshold (*q*-value = 0.25). A: basal; C: end of therapy.
