# Supplementary material for: Dissecting Molecular Heterogeneity of Circulating Tumor Cells (CTCs) from Metastatic Breast Cancer Patients through Copy Number Aberration (CNA) and Single Nucleotide Variant (SNV) Single Cell Analysis
Source: Cancers (Basel). 2022 Aug 14;14(16):3925. doi: 10.3390/cancers14163925 (PMC9405921; doi:10.3390/cancers14163925)
Supplement: Supplementary file 1 [file cancers-14-03925-s001.zip › Supplementary Material Table S1.pdf]

Supplementary Table S1. List of the variants detected in single circulating tumor cells (CTCs) of patients in the different timepoints. Time A: baseline; time B: 6-8 weeks after the beginning of treatment; time C: 2-4 weeks after the last dose of therapy.

Patient CH29	**Time A**										
	1	4	5	6	8	12	14	15	17	18	
	APC c.4451A>G<br>p.D1484G	*ATM* c.998C>T<br>p.S333F	*ATM* c.998C>T<br>p.S333F	*ATM* c.998C>T<br>p.S333F	*ATM* c.998C>T<br>p.S333F	*ATM* c.998C>T<br>p.S333F	*ATM* c.998C>T<br>p.S333F	*ATM* c.998C>T<br>p.S333F	*ATM* c.998C>T<br>p.S333F	*ATM* c.998C>T<br>p.S333F	
		AKT1 c.63delC<br>p.T21fs	*NOTCH1* c.7327G>T<br>p.V2443L		*TP53* c.628C>T<br>p.R210X	*TP53* c.628C>T<br>p.R210X	*EGFR* c.872G>A<br>p.C291Y		*EGFR* c.2092G>A<br>p.A698T		
						PTEN c.900C>T<br>p.I300I			*SMAD4* c.1275delT<br>p.A425fs		
									PDGFRA c.2558G>A<br>p.G853D		
									SMAD4 c.529C>A<br>p.H177N		
	**Time B**										
	2	8	9								
	ATM c.998C>T<br>p.S333F	*ATM* c.998C>T<br>p.S333F	*TP53* c.628C>T<br>p.R210X								
	PTEN c.860C>G<br>p.S287X	*PTEN* c.860C>G<br>p.S287X	*CDKN2A* c.346G>T<br>p.D116Y								
	**Time C**										
	4	20									
	ATM c.998C>T<br>p.S333F	*FLT3* c.1361C>A<br>p.S454X									
	FGFR1 c.545G>A<br>p.S182N	*APC* c.3067C>A<br>p.Q1023K									
	RB1 c.2264T>C<br>p.F755S										
Patient CH30	**Time A**										
	1	5	8	4bis	5bis	8bis	15	16	17	15bis	18bis
	MET c.2947C>A<br>p.L983I	*GNA11* c.694C>A<br>p.L232I	*TP53* c.678G>T<br>p.E226D	*RET* c.2342A>C<br>p.Q781P	*VHL* c.407delG<br>p.R136fs	*NRAS* c.196G>T<br>p.A66S	*ABL* c.1057G>T<br>p.E353X	*TSC1* c.1444C>T<br>p.P482S	*JAK3* c.418C>T<br>p.Q140X	*ABL1* c.772C>A<br>p.R258S	*PIK3CA* c.1033A>G<br>p.N345D
	ALK c.3778C>T<br>p.P1260S								*ERBB2* c.935C>T<br>p.T312I		*VHL* c.411delG<br>p.L137fs
	MLH1 c.380C>T<br>p.S127L										*BRAF* c.1372A>G<br>p.T458A
	**Time C**										
	1	2									

	HNF1A c.851A>G p.D284G	*JAK3* c.406C>T p.H136Y		
	PIK3CA c.2699C>G p.S900X	*FBXW7* c.1122G>T c.G1122T		
	**Time A**			
	1	2	5	1BIS
Patient CH32	*HNF1A* c.893C>T p.A298V	*AKT1* c.49G>A p.E17K	*AKT1* c.49G>A p.E17K	*TP53* c.73G>T p.V25F
	TP53 c.518A>G p.K173R	*TP53* c.384delC p.S128fs	*TP53* c.384delC p.S128fs	*SMO* c.608C>T p.P203L
			RB1 c.1032G>T p.Q344H	
